# Glyphosate Resistance of C_3_ and C_4_ Weeds under Rising Atmospheric CO_2_

**DOI:** 10.3389/fpls.2016.00910

**Published:** 2016-06-22

**Authors:** Nimesha Fernando, Sudheesh Manalil, Singarayer K. Florentine, Bhagirath S. Chauhan, Saman Seneweera

**Affiliations:** ^1^School of Applied and Biomedical Sciences, Faculty of Science and Technology, Federation University, Mount Helen Campus, Ballarat, VICAustralia; ^2^School of Plant Biology, UWA Institute of Agriculture, The University of Western Australia, Crawley, WAAustralia; ^3^Amrita University, CoimbatoreIndia; ^4^The Centre for Plant Science, Queensland Alliance for Agriculture and Food Innovation, The University of Queensland, Toowoomba, QLDAustralia; ^5^Centre for Crop Health, University of Southern Queensland, Toowoomba, QLDAustralia

**Keywords:** glyphosate resistance, C_3_ weeds, C_4_ weeds, elevated [CO_2_], photosynthesis strategies, weed competition

## Abstract

The present paper reviews current knowledge on how changes of plant metabolism under elevated CO_2_ concentrations (e[CO_2_]) can affect the development of the glyphosate resistance of C_3_ and C_4_ weeds. Among the chemical herbicides, glyphosate, which is a non-selective and post-emergence herbicide, is currently the most widely used herbicide in global agriculture. As a consequence, glyphosate resistant weeds, particularly in major field crops, are a widespread problem and are becoming a significant challenge to future global food production. Of particular interest here it is known that the biochemical processes involved in photosynthetic pathways of C_3_ and C_4_ plants are different, which may have relevance to their competitive development under changing environmental conditions. It has already been shown that plant anatomical, morphological, and physiological changes under e[CO_2_] can be different, based on (i) the plant’s functional group, (ii) the available soil nutrients, and (iii) the governing water status. In this respect, C_3_ species are likely to have a major developmental advantage under a CO_2_ rich atmosphere, by being able to capitalize on the overall stimulatory effect of e[CO_2_]. For example, many tropical weed grass species fix CO_2_ from the atmosphere via the C_4_ photosynthetic pathway, which is a complex anatomical and biochemical variant of the C_3_ pathway. Thus, based on our current knowledge of CO_2_ fixing, it would appear obvious that the development of a glyphosate-resistant mechanism would be easier under an e[CO_2_] in C_3_ weeds which have a simpler photosynthetic pathway, than for C_4_ weeds. However, notwithstanding this logical argument, a better understanding of the biochemical, genetic, and molecular measures by which plants develop glyphosate resistance and how e[CO_2_] affects these measures will be important before attempting to innovate sustainable technology to manage the glyphosate-resistant evolution of weeds under e[CO_2_]. Such information will be of essential in managing weed control by herbicide use, and to thus ensure an increase in global food production in the event of increased atmospheric [CO_2_] levels.

## Introduction

Chemical weed management with use of herbicides is the most economical and widely used weed management technique in the world. This is mainly because of their potential to improve crop yields and save labor and energy, thereby reducing the cost of farming. Among the herbicides, glyphosate, a non-selective and post-emergence herbicide, is the most widely used agent in global agriculture since its introduction in 1974 (Duke and Powles, 2008). Its ability to control a broad range of weeds has made it the world’s most important herbicide (Baylis, 2000; Powles, 2008). Moreover, introduction of transgenic glyphosate-resistant crops has led to large increase of usage of glyphosate around the world (James, 2010).

Herbicide action generally depends on a metabolic function (growth, amino acid synthesis, photosynthesis, lipid synthesis, etc.) that is essential for plant growth and development. Consequently, any anatomical, morphological, metabolic, and/or physiological changes in plants due to predicted climate change can also affect the performance of herbicides. At present, climate change has been recognized as one of the global issue, thus, understanding of how plants respond to climate variables and the resulting effects on the efficacy of glyphosate is the priority in terms of agriculture productivity and sustainability. Among the key climate variables, rising atmospheric CO_2_ concentration ([CO_2_]) is recognized as one of the critical component involved in climate change. Global [CO_2_] is continuously increasing and will reach 550 mol mol^-1^ by 2050, according to the IPCC (Intergovernmental Panel on Climate Change) emission scenario A1B (Carter et al., 2007). Over the last two decades, the amount of CO_2_ available to plants has significantly increased from a preindustrial era concentration of 280–400 ppm in 2014. Such a large change in atmospheric [CO_2_]; a key resource for plant growth and development will have significant impact through adjusting anatomical, morphological, metabolic, and physiological properties of the plants (Ainsworth and Long, 2005; Taub et al., 2008). Hence, these changes in plants are likely to influence the herbicide uptake, transport, metabolism, and overall effectiveness of glyphosate.

Elevated atmospheric [CO_2_] (e[CO_2_]) also facilitate the increase in mean global temperature (Houghton, 2001) leading to climate change, particularly more frequent drought and heat waves (Mpelasoka et al., 2008). However, many weed species have well adapted to climate stresses such as heat and drought through development of biochemical and physiological strategies (Patterson and Flint, 1990). Many adaptive strategies have been identified either to avoidance and/or acclimate to cope with heat and drought stresses. Some of the examples for those strategies are accumulation of antioxidants, osmoprotectants, heat shock proteins, signaling cascades, and transcriptional control which involved in offsetting stress-induced biochemical and physiological alterations (Hasanuzzaman et al., 2013). Consequently, weed species are likely to out-compete main crop species for the use of available resources under changing climate conditions. Competition between weed species and crops has been shown to be dependent on [CO_2_] richness of the atmosphere (Patterson and Flint, 1990). In particular, the magnitude of growth and yield response to e[CO_2_] varies among plant functional groups (Taub et al., 2008), and the growth rate also depends on air temperature, soil nutrient, and water interactions (Kimball et al., 2002). Therefore, the benefits of raising atmospheric [CO_2_] are not even for all species, and those weedy species that take greater advantage due to e[CO_2_] are likely to be a major challenge for crop productivity in the twenty-first century. Among major photosynthetic traits, C_3_ species benefit from e[CO_2_] mainly through their ability to improve photosynthetic capacity and water use efficiency (Ainsworth and Long, 2005), whilst in the case of C_4_ species, they can take advantage only of improved water use efficiency. Water use efficiency of both C_3_ and C_4_ species improved due to decreased stomatal conductance under e[CO_2_] (Ainsworth and Long, 2005). Thus, overall, C_3_ species are likely to become a major competitor under [CO_2_]-rich atmosphere. For example, the CO_2_ fixation mechanism of C_4_ plants has been shown to be saturated at 360 ppm making them less likely to show any increased photosynthesis response to e[CO_2_] (Leegood, 2002). It is known that more than 95% of plant species belong to C_3_ family, and thus the major C_3_ crop plants could have a competitive advantage over many of the C_4_ weed species because they have been selectively bred for yield. These species, especially C_3_ crops maintain large degree of genetic homogeneity and thus glyphosate resistance can be rapidly developed. However, there is yet another complicating factor which must be taken into account before we can fully understand the effects of e[CO_2_]. This factor relates to the unknown effect of e[CO_2_] on herbicide efficacy, which may come into play to both limit and lower the initially expected competitive advantage of the C_3_ crop species (Ziska et al., 1999).

Widespread glyphosate usage with higher concentrations than the recommended levels of glyphosate in many parts of the world has led to evolution of glyphosate resistance in both C_3_ and C_4_ weed types (Heap, 2013). Today, modern crop production has all too often abandoned diverse crop rotations and tillage, trading them for monoculture crops and no till/low till technologies which rely on an effective herbicide technology. As a result, after several decades of concentrated herbicide use, weed resistance to major chemical classes continues to spread further. Currently more than 60% of the global herbicide market in terms of value is represented by products from only four modes of action [5-enolpyruvylshikimate-3-phosphate synthase (EPSPS), acetolactate synthase, Auxins, and ACCase, acetyl-CoA carboxylase], all of which actually have serious resistance issues (Kraehmer et al., 2014). Plants adapt to inevitable e[CO_2_] through changing several metabolic and physiological processes (mainly carbon and nitrogen metabolism, plant hormonal metabolism, and plant water relations) which are likely to affect (i) weed competitiveness with crops, (ii) the level of herbicide efficacy (Ziska and Dukes, 2011), and (iii) the related increase of herbicide resistance in some weeds. Therefore, it is important to understand how e[CO_2_] influence the development of glyphosate resistance in plants that utilize C_3_ and C_4_ photosynthetic pathways. This understanding is essential to management of weeds in changing climatic conditions in the future. The present paper will therefore focus on how e[CO_2_] affect the glyphosate resistance in C_3_ and C_4_ weeds.

## A Brief Overview of Glyphosate Resistance in Plants

Glyphosate [*N*-(phosphonomethyl)-glycine] inhibits EPSPS, an enzyme responsible for the synthesis of aromatic amino acids (Steinrucken and Amrhein, 1984). A non-selective broad spectrum weed control, systemic herbicidal action, lack of soil residual activity, low mammalian toxicity, and rapid soil biodegradation are some of the factors that favored the wide acceptability of glyphosate (Duke and Powles, 2008; Powles, 2008; Preston et al., 2009). The patent on glyphosate expired in 2000, which resulted in multiple producers entering in to the global market, driving cheap competitive pricing which has resulted in significantly increased adoption (Pollegioni et al., 2011). Herbicide resistance in glyphosate was not reported in the first 20 years of its introduction (in 1974), however, there now have been an alarmingly increasing number of resistant cases reported from many parts of the world (Sammons and Gaines, 2014). At present, 31 weed species have been reported to be glyphosate resistant from different agro-ecosystems (Heap, 2013). It appears that over-reliance on this herbicide, which is manifested in actions such as herbicide use without diversity and a general lack of integration with other herbicides, cultural and biological methods, have contributed to the rapid evolution of glyphosate-resistant weeds (Powles, 2008; Preston et al., 2009; Shaner et al., 2012). Most importantly, coupled with the previous factors, increased adoption of glyphosate tolerant crops have been significantly contributing to the evolution of glyphosate-resistant weeds (Powles, 2008; Preston et al., 2009; Beckie and Hall, 2014). The enormity of this problem is illustrated by the increase in area under genetically modified crops; this has increased from 1.7 million hectares in 1996 to over 175 million hectares in 2013, of which a major portion is glyphosate-resistant crops (Jmaes, 2013). Interestingly, 19 developing countries have now begun to grow genetically modified crops (Jmaes, 2013) and therefore the adoption of glyphosate-resistant crops and intensive glyphosate usage is likely to be further enhanced.

## Glyphosate Resistance Mechanisms

The development of herbicide-resistance is generally classified as either “target site” or “non-target site” mechanisms (Yuan et al., 2007). In the case of a “target site” mechanism, a weed can acquire resistance to herbicides by a mutation in the gene coding for the enzyme targeted by the herbicide. In herbicide-resistant biotypes, the amino acid Pro, at site 106, has been substituted by a Ser, Thr, Ala, or Leu amino acid which alters the ability of glyphosate to bind to the enzyme EPSPS (Powles, 2008; Preston et al., 2009; Perez-Jones and Mallory-Smith, 2010; Sammons and Gaines, 2014). This target site resistance was reported in many field-evolved resistant weed populations, including *Eleusine indica* (L.) Gaertn. (Ng et al., 2003), *Lolium* sp. (Perez-Jones et al., 2007; Kaundun et al., 2011; Bostamam et al., 2012; Collavo and Sattin, 2012), *Digitaria insularis* (L.) Mez ex Ekman (de Carvalho et al., 2012), *Echinochloa colona* (L.) Link. (Alarcon-Reverte et al., 2013), and *Amaranthus tuberculatus* L. (Nandula et al., 2013). Having this target site resistance mechanism imparts a 2- to 10-fold resistance over susceptible biotypes (Wakelin and Preston, 2006). The target site mechanism is widely exploited for the synthesis of glyphosate-resistant crop plants, where transgenic crops contain a gene derived from *Agrobacterium* sp. strain CP4. This synthesis codes in the glyphosate-tolerant enzyme, CP4 EPSPS, which confers glyphosate tolerance in crop plants (Padgette et al., 1995).

The resistance through “non-target site” mechanisms could be due to a number of factors, including rapid metabolism of the herbicide, sequestration of herbicide in a vacuole, reduced translocation, or reduced leaf uptake (Yuan et al., 2007). Although the metabolism of herbicide into non-toxic substrates is a major non-target site resistance mechanism, none of the glyphosate-resistant weed populations screened have been found to possess this mechanism. In most cases, reduced translocation has conferred glyphosate resistance in many evolved glyphosate-resistant populations including, *A. tuberculatus* (Nandula et al., 2013), *Lolium* sp. (Wakelin et al., 2004; Bostamam et al., 2012), *D. insularis* (de Carvalho et al., 2012), *Conyza canadensis* (L.) Cronq. (Ge et al., 2010), and *Sorghum halepense* (L.) Pers. (Vila-Aiub et al., 2012). The evolved resistances through these mechanisms are at a similar or higher level than those involved in target site mechanisms (Preston et al., 2009; Sammons and Gaines, 2014). The EPSPS enzyme is highly expressed and produced in the growing meristems of both root and shoot (Shaner, 2009), therefore, to be effective, glyphosate has to travel from the leaf to other growing parts of the plant. In resistant biotypes, this movement of glyphosate is restricted in leaves (Shaner, 2009; Sammons and Gaines, 2014). Vacuolar sequestration is identified as the major underlying mechanism that endows glyphosate resistance (Ge et al., 2010; Mohseni-Moghadam et al., 2013; Sammons and Gaines, 2014). In addition, reduced leaf uptake has been identified as a mechanism, leading to glyphosate resistance in weeds (Vila-Aiub et al., 2012; Sammons and Gaines, 2014). In addition, soil bacteria could also contribute to metabolize glyphosate (Kishore and Jacob, 1987; Sviridov et al., 2015). It has been suggested that high level of glyphosate resistance can achieved by transferring these metabolizing genes into crop plants (Pline-Srnic, 2006). However, evolved resistance due to glyphosate metabolism is not observed in weeds (Pline-Srnic, 2006).

A new mechanism, gene amplification, was identified behind glyphosate-resistant strain which had evolved in *Amaranthus palmeri* L. (Gaines et al., 2010, 2011). The resistant *A. palmeri* genomes contained many more copies of the EPSPS gene (up to 160-fold) than the susceptible plants (Gaines et al., 2010, 2011). In this way, glyphosate molecules could bind only a portion of EPSPS molecules, thereby the enzyme functionality would be maintained in the resistant plants. The herbicide resistance afforded by gene amplification is much more effective compared to the other non-target site mechanisms (Gaines et al., 2010). Gene amplification is hypothesized to be mediated by transpose or RNA-mediated mechanisms (induced by stress), followed by herbicide selection and enrichment in the weed population (Gaines et al., 2010, 2011). Gene amplification and the increased activity of the EPSPS enzyme were both reported in *Amaranthus spinosus* L. (Nandula et al., 2014) and *Lolium* populations (Salas et al., 2012; Nandula et al., 2014). These mechanisms could confer very high level of resistance, depending upon the level of gene amplification and expression of EPSPS genes (Sammons and Gaines, 2014).

In many instances, weed populations were found to possess a combination of the target site and non-target site mechanisms, leading to an enhanced level of glyphosate resistance (Vila-Aiub et al., 2012; Nandula et al., 2013; Sammons and Gaines, 2014). This has evolutionary implications, especially in cross-pollinated weed species where resistance mechanisms rapidly spread due to pollen movement. However, not all mechanisms impart a very high level of resistance; as an example, Leu substitution at Pro 106 site imparts only 1.7-fold resistance in *Lolium rigidum* Gaud. Intentional application of low herbicide dosages may have the potential to select such mutations (Busi and Powles, 2009; Manalil, 2014).

## Increasing Atmospheric CO_2_ on Glyphosate Resistance

The effectiveness of any applied herbicide on weed control is influenced by the amount applied, the timing of the application relative to the biology of weed, and the environmental conditions during and after application (Ziska and Dukes, 2011). Environmental factors such as soil moisture, rainfall, wind, relative humidity, soil and air temperature, and light can also influence the herbicide efficacy. It has also been demonstrated that increased atmospheric [CO_2_] also influence on the herbicide efficacy (Ziska et al., 1999). Here, we discuss the process of how e[CO_2_] will affect the glyphosate resistance development in both C_3_ and C_4_ plants based on biochemical, physiological, metabolic, and morphological differences in plants that can be occurred under increased atmospheric [CO_2_] in different levels of functional organization scaling from the cellular to organism level.

## Biochemical Level

**FIGURE 1:**
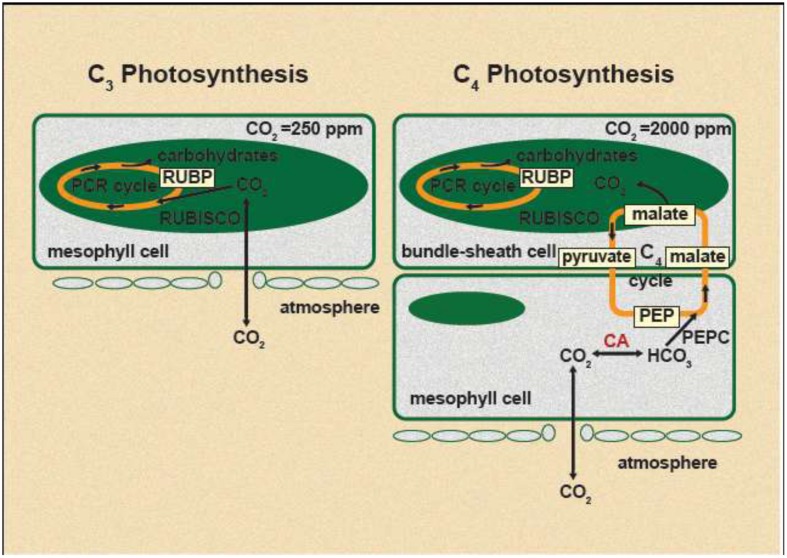
**Schematic representation of C_3_ and C_4_ photosynthesis under current atmospheric CO_2_ concentration.** Approximate intercellular CO_2_ concentration in C_3_ species is 250 ppm and in C_4_ species is 2000 ppm (Yoshie, 1986).

C_3_ and C_4_ plants are basically different in their biochemical process of photosynthetic pathways. The majority of major crop species and ~85% of terrestrial plants use C_3_ photosynthesis, while C_4_ crops are a minority, represented predominantly by maize, sorghum, and sugarcane among economically important crops (Ehleringer et al., 1997). However, many of tropical weed grass species fix CO_2_ from the atmosphere via the C_4_ photosynthetic pathway, a complex anatomical and biochemical variant of the C_3_ pathway (**Figure 1**; Hatch, 1987). C_4_ photosynthesis was evolved as an adaptation to high light intensities, high temperatures, dryness, and low atmospheric [CO_2_]. Therefore, C_4_ plants dominate grassland floras and biomass production in the warmer climates of the tropical and subtropical regions (Edwards et al., 2010).

Glyphosate resistance has been reported previously with many C_3_ plant species (Ziska and Dukes, 2011). In recent past, increased glyphosate resistance under e[CO_2_] has been reported in noxious C_3_ species such as Canada thistle (*Cirsium arvense*; Ziska et al., 2004). Photosynthetic rate of C_3_ plants approximately doubled when plants grown at about 380 μmol CO_2_ mol^-1^ were exposed to 700 μmol CO_2_ mol^-1^ (Ainsworth and Rogers, 2007). In general, C_3_ plants use ATP and NADPH generated by light reaction of photosynthesis to fix atmospheric CO_2_ directly via ribulose bisphosphate carboxylase–oxygenase (RuBisCO; **Figure 1**). Because of the dual activity of RuBisCO (carboxylation and oxygenation), both CO_2_ and O_2_ compete for the same site on RuBisCO, resulting in a 20–60% loss of substrate (CO_2_) through photorespiration (oxygenation) under current atmospheric [CO_2_] (Bowes et al., 1996). e[CO_2_] increase the ratio of CO_2_:O_2_ at the site of CO_2_ fixation in the chloroplast of C_3_ plants, which favor the photosynthetic carbon reduction (PCR) cycle over photorespiratory carbon oxidation cycle leading to higher photosynthesis rate (Seneweera et al., 2005). Under normal growth conditions, about 20% of total photosynthetically fixed carbon is estimated to be consumed by the shikimate pathway (Herrmann and Weaver, 1999). Under e[CO_2_], due to stimulation of light-saturated photosynthesis, more carbon intermediates available for other essential pathways, such as synthesis of more EPSPS enzyme. It has been found that EPSPS copy number is higher in glyphosate resistant plants relative to the susceptible plants (Gaines et al., 2010; Mohseni-Moghadam et al., 2013). Moreover, it has been shown that shikimate accumulation is very low in glyphosate resistant *A. palmeri* plants which has higher EPSPS copy number, compared to the glyphosate sensitive plants (Gaines et al., 2010; Mohseni-Moghadam et al., 2013). Therefore it is worthwhile to study whether synthesis of EPSPS will increase under e[CO_2_] due to availability of more carbon intermediates than the current level of atmospheric [CO_2_], which can directly impact on glyphosate resistant development.

**FIGURE 2:**
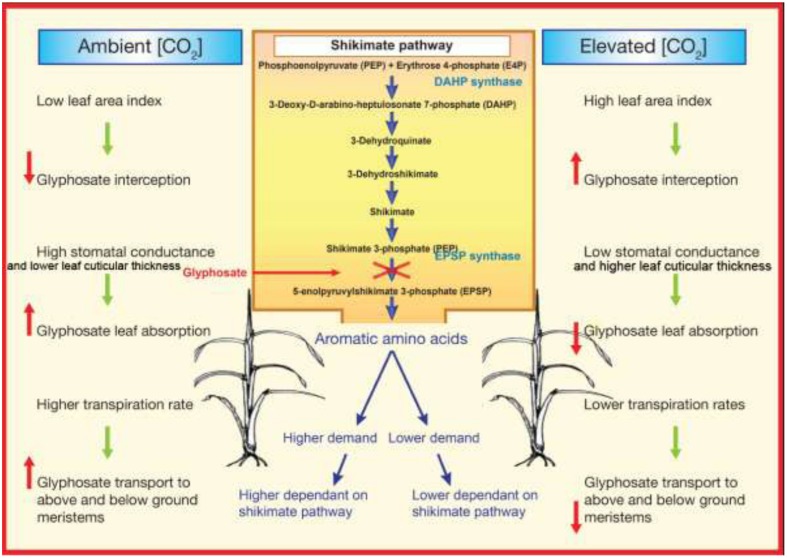
**Schematic representation of why glyphosate resistant development is possibly easier under elevated atmospheric CO_2_ concentrations than under ambient atmospheric CO_2_ concentrations**.

The primary mechanism, by which plant growth accelerates at e[CO_2_], is through an increase in photosynthetic rates as a result of increase in RuBisCO carboxylation efficiency (Bowes et al., 1996; Drake, 1997; Nakano et al., 1997). Increase carboxylation of RuBisCO at e[CO_2_] lead to reduce the RuBisCO content, particularly in C_3_ plants. In general, photosynthetic and photorespiratory enzymes represent a large proportion of leaf N which is more than 25% (Makino and Mae, 1999). For example, RuBisCO content can decreases up to 30% when plants are exposed to e[CO_2_] for extended period (Stitt and Krapp, 1999; Gifford et al., 2000). Decreased RuBisCO content under e[CO_2_], results a decline of leaf N status (Stitt and Krapp, 1999) and then the plant protein synthesis. In a recent meta analysis of 122 studies on effect of e[CO_2_] on leaf nutrient concentration has been shown that total leaf N (by 16%), amino acids (by 14%) and soluble proteins (by 10%) decreased by e[CO_2_], while subsequently increased of starch content (Robinson et al., 2012). Moreover, in recent studies it has been shown that N concentration of leaves (Högy et al., 2009) and in grains (Högy et al., 2009; Fernando et al., 2012a,b, 2014, 2015) were significantly decreased under e[CO_2_]. Less protein in plant tissues could, in turn, results in less demand for aromatic and branch amino acids (**Figure 2**). Amino acids are essential building blocks for protein synthesis. Proteins are required for the production of new cells and for the functioning of many plant processes. Plants synthesize amino acids from carbohydrates (produced by means of photosynthesis) and nitrogen through collateral metabolic pathways. Any change in the environmental conditions that affect carbohydrate production through photosynthesis or nitrogen content in the plants can alter amino acids production, subsequently affecting the efficacy of herbicides. Less protein may lead to less demand for amino acids synthesis, which in turn may alter the efficacy of amino acids inhibitors. Currently there is a strong evidence that glyphosate action site is located at EPSPS enzyme (Schönbrunn et al., 2001; Wakelin and Preston, 2006), confirmed by a subsequent decrease in synthesis of aromatic amino acids tryptophan, phenylalanine, and tyrosine. Many assume that glyphosate kill plants through inhibition of EPSPS, which leads an insufficient aromatic amino acids production to maintain essential proteins synthesis that has rapid rates of turnover (Powles, 2008; Mohseni-Moghadam et al., 2013). Therefore, it is worthwhile to conduct studies to determine whether lower the aromatic amino acids demand in C_3_ plants under e[CO_2_], may lead to development of glyphosate resistance under e[CO_2_].

C_4_ plants are photosynthetically saturated at the current [CO_2_] level and rising atmospheric [CO_2_] would have no major impact on their C fixation rate, biomass production, and yield (Ainsworth and Long, 2005). It has been demonstrated that e[CO_2_] had a little (Zhang et al., 2015) or no effect on growth of goosegrass (*E. indica*), a C_4_ species under favorable conditions (Patterson, 1986). Moreover, Zhang et al. (2015) has shown that in goosegrass, glyphosate resistant biotypes decreased the glyphosate resistance by 60% when exposed to e[CO_2_] while glyphosate resistance slightly increased in susceptible biotypes. In C_4_ plants, atmospheric CO_2_ is fixed in the bundle sheath cells by RuBisCO after a primary fixation in to C_4_ compounds by phosphoenolpyruvate carboxylase (PEPC) in mesophyll chloroplast (Hatch, 1987; **Figure 1**). These C_4_ compounds are transported to the bundle sheath cells and decarboxylated to yield CO_2_ which is then assimilated by RuBisCO through PCR cycle (**Figure 1**; Hatch, 1987). The C_3_ compounds that produced through the decarboxylation reaction return to the mesophyll cells to regenerate phosphoenolpyruvate (PEP) in a reaction catalyzed by the enzyme pyruvate orthophosphate dikinase. It has been reported that glyphosate resistant goosegrass biotypes consistently showered with lower concentration of PEPC, an enzyme involve in the C assimilatory pathway. It was shown that when low PEPC, there are more N and P resources are available for EPSPS gene amplification in resistant biotypes (Zhang et al., 2015). However, it is not known how e[CO_2_] impact on PEPC content in C_4_ plants and hence glyphosate resistant development.

Even though, the above explained CO_2_ fixing general scheme is common among the C_4_ species; there are variations to this basic pathway that include diverse decarboxylation enzymes as well as different metabolites transport systems. Decarboxylation process occurs in three diverse ways in C_4_ species, mainly using one of the following enzymes: NADP-malic enzyme (NADP-ME), NAD malic enzyme (NAD-ME) or phosphoenolpyruvate carboxykinase (PEP-CK). Therefore, based on the enzyme catalyzing the decarboxylation reaction of C_4_ acids, C_4_ plants have been grouped into three biochemical subtypes (C_4_/NADP-ME, C_4_/NAD-ME, and C_4_/PEP-CK; Leegood and Walker, 1999; Edwards et al., 2001). Each C_4_ subgroup possesses particular structural features, biochemistry and physiology, and also differences in the mechanism used to regenerate PEP, the substrate of PEPC in mesophyll cells. Nevertheless, it is now becoming apparent that, in several C_4_ plants, more than one decarboxylase operates at the same time (Drincovich et al., 2011). C_4_ species respond differentially to increasing atmospheric CO_2_ in-terms of glyphosate tolerance (Manea et al., 2011). Manea et al. (2011) and his group showed that three of the four C_4_ grass species that they treated with the recommended concentration of glyphosate (*Chloris gayana*, *Eragrostis curvula*, *Paspalum dilatatum* Poir) increased the glyphosate tolerance, while remained C_4_ grass species *Sporobolus indicus* did not change glyphosate tolerance under e[CO_2_]. For example, *C. gayana* and *S. indicus* species are belong to C_4_/PEP-CK biochemical type, *E. curvula* is belong to C4/NAD-ME biochemical type (Ueno et al., 2005) while *P. dilatatum* Poir is a C4/NADP-ME type (Cavaco et al., 2003). Despite, *C. gayana* and *S. indicus* species belongs to C_4_/PEP-CK biochemical type, *S. indicus* shows a significant higher hydroxypyruvate reductase and catalase activity compared to the other three species (Ueno et al., 2005).

These findings suggest that photosynthetic biochemistry together with expression level and activity of photorespiratory enzymes within C_4_ sub types plays a key role in determining glyphosate resistance. However, Manea et al. (2011) demonst rated that the different response to glyphosate tolerance of these C_4_ species are associated with relative [CO_2_] response, in terms of biomass production under e[CO_2_]. These findings are purely based on the plant morphological observations, but underlying glyphosate resistance is likely to drive through both morphological and biochemical adjustments at cellular to whole plant level.

Moreover, regardless to e[CO_2_] mediated glyphosate metabolism, Roso and Vidal (2010) have been proposed a modified phosphate carrier protein theory by integrating six hypotheses together to explain the glyphosate resistant under current growing conditions. After glyphosate absorption occurs, it would normally move with the transpiration flow and would be loaded into the phloem in order to reach the site of action. It has been speculated that an unidentified barrier that prevents glyphosate entry into the phloem and/or the presence of some carrier protein that impedes the presence of the glyphosate on the sensitive tissue results in lack of phloem loading of the herbicide in resistant weeds.

The proposed different hypotheses to explain the mechanism of reduced translocation of glyphosate in resistant biotypes were, (a) change in the transporter that carries the glyphosate into the cell; (b) increased action of a transporter that carries the glyphosate into the vacuole; (c) increased active eﬄux pumps of glyphosate; (d) increased action of a transporter that carries glyphosate out of the chloroplast (Shaner, 2009) and hypotheses to explain the lack of glyphosate inside the chloroplast; (e) reduced movement of the herbicide through the transpiration flow; and (f) inability of the herbicide to re-enter the phloem (Roso and Vidal, 2010). Therefore, it is possible that glyphosate resistance in C_4_ plants may be associated with photosynthetic compartmentation. As in C_4_ plants, [CO_2_] is primary fixed in mesophyll cells and C_4_ product transported to bundle sheath cells for its decarboxylation and [CO_2_] fixation, these processes may involve discrimination of other metabolite transfer between mesophyll cells and bundle sheath cells. To permit the efficient function of C_4_ photosynthesis mesophyll and bundle sheath cells are tightly interconnected to each other by high numbers of plasmodesmata (Dengler and Nelson, 1999). Therefore, it is possible that glyphosate transfers between mesophyll cells to bundle sheath cells is lower in C_4_ plants and consequently reduce its transport between sources to sink tissues. This possibly increase the development potential of glyphosate resistant in C_4_ weeds under e[CO_2_] according to the suggested modified phosphate carrier protein theory.

### Leaf Level

Glyphosate is a water soluble herbicide that penetrates through stomata and leaf cuticle (Dewey and Appleby, 1983; Preston and Wakelin, 2008). Electrophysiological studies showed that e[CO_2_] increases the activity of outward rectifying K^+^ channels, decreases the activity of inward rectifying K^+^ channels, enhances S type anion channel activities, stimulates Cl^-^ release from stomatal guard cells and increases guard cell Ca^2+^ concentration (Webb et al., 1996). These changes collectively depolarize the membrane potential of guard cells and cause stomatal closure (Assmann, 1993). Therefore, greater depolarization at e[CO_2_] will result in a reduced stomatal conductance. Decreased stomatal conductance and number may reduce penetration of glyphosate in to plants by which reduce the efficacy of glyphosate (**Figure 2**; Ziska and Teasdale, 2000). However, glyphosate absorption is not only through the stomata, it also taken up through the outer cuticle layer. It has been reported that plants grown under e[CO_2_] increase the leaf cuticular thickness and leaf pubescence in both C_3_ and C_4_ plants (Ainsworth and Long, 2005). These anatomical changes may contribute to decrease glyphosate penetrates through the leaf cuticle hence decreased glyphosate efficacy under e[CO_2_] (**Figure 2**). For example, long-term exposure (231 days) to e[CO_2_] of 720 μmol mol^-1^ increased glyphosate tolerance in quackgrass (*Elymus repens*) compared to plants grown under ambient [CO_2_]. It was speculated that this response could have been associated with either a reduction in stomatal conductance that may have decreased glyphosate absorption or high starch concentrations within the chloroplast that reduce glyphosate diffusion to specific sites (Ziska and Teasdale, 2000). Similar to C_3_ plants, stomatal conductance in C_4_ plants are consistently reduced under e[CO_2_] (Ainsworth and Long, 2005). However, for C_4_ plants, CO_2_ fixation is saturated at 360 ppm, so these plants are less likely to show a positive response to additional [CO_2_] availability (Leegood, 2002). This makes predicting the response of C_4_ plants to e[CO_2_] more difficult (Dukes, 2000). This unpredictability is demonstrated by studies that show the growth of C_4_ plants can be stimulated (Bazzaz et al., 1989; Owensby et al., 1999), not affected (Erickson et al., 2007), or inhibited (Belote et al., 2003) under e[CO_2_].

Plant growth response to e[CO_2_] is mainly characterized by accelerated leaf area production, resulting in higher relative growth rate and then higher leaf area ratio (Nakano et al., 1997). This early advantage at e[CO_2_] results from faster leaf blade elongation rate, which leads to greater leaf area, biomass production, and grain yield (Conroy et al., 1994; Jitla et al., 1997). Leaf elongation rate is a function of rates of epidermal cell division, elongation, and the duration of individual cells (Skinner and Nelson, 1995). In general, increased leaf area may increase the interception of post-emergent herbicides such as glyphosate. Leaf area development response is less than biomass respond to e[CO_2_] in C_3_ weeds (Ainsworth and Long, 2005) while leaf area and biomass responses to e[CO_2_] are almost similar in C_4_ weeds (Ainsworth and Long, 2005). Differential responses of leaf area and total biomass between C_3_ and C_4_ weeds may have a differential effect of glyphosate efficacy in [CO_2_] enriched world.

### Whole Plant Level

Growth of both C_3_ and C_4_ weeds will directly affect by e[CO_2_] (Ainsworth and Long, 2005). In a meta-analysis, which summarized the response to increasing [CO_2_] showed that mean biomass response to e[CO_2_] is greater in C_3_ weed species (130%: 33 species) than in C_4_ weed species (115%: 11 species). For example, lambsquarters (*Chenopodium album*; a C_3_ species) which showed a higher tolerance to glyphosate spend about 5 days less in the seedling stage and also increased growth and biomass when grown under e[CO_2_] (Ziska et al., 1999). Decreased length of seedling stage under e[CO_2_], which is the greatest herbicide sensitive stage of the plants, may reduce the glyphosate efficacy at e[CO_2_]. Glyphosate application timing could be adjusted to apply earlier under e[CO_2_] than the current timing to escape this scenario.

It has been reported field-grown Canada thistle increased growth and root:shoot ratio under e[CO_2_], which resulted in the reduced efficacy of glyphosate because of the dilution effect caused by large stimulation of below-ground growth Ziska et al. (2004). These challenges can be easily overcome by higher application rates under e[CO_2_], on other hand, this will increase the cost of weed management. There has been insufficient data on the role of herbicide dose on resistance development even under current climate conditions (Manalil, 2014). High herbicide dose results in high level of mortality but select for rare resistance genes which has the capability of endowing high-level resistance (Neve and Powles, 2005). Similarly, it has been reported that sub-lethal dose (where some plants are killed and some plants are survive), also results in the accumulation of minor genes with additive or multiplicative effects which could lead to polygenic traits herbicide resistance development in the field (Manalil, 2014).

Upon entering to the plant, glyphosate is translocated symplastically via phloem to apical meristem follows photoassimilate translocation from source to sink. Glyphosate translocation follows source-to-sink patterns similar to photosynthates, suggesting that phloem translocation plays a major role. It has been reported that glyphosate moves symplastically from sources tissue toward sinks tissues, but not necessarily in the same proportions as assimilates (Dewey and Appleby, 1983). However, it has been reported that glyphosate translocate via xylem as well and apoplast movement plays a major role in determining overall glyphosate distribution within a plant, especially when an application method results in any deposition of glyphosate on the stems (Dewey and Appleby, 1983). As photosynthesis rate is greater under e[CO_2_] than the current atmospheric[CO_2_], assimilates transfer between source to sink tissue is expected to be greater under e[CO_2_]. The ability of glyphosate to control perennial plants is a result of the extensive translocation of glyphosate from its site of application, usually the leaves, to all parts of the plant including the roots. Consistent with lower stomatal conductance lower transpiration rates are commonly reported in CO_2_ enrichment studies (Long et al., 2004; Ainsworth and Rogers, 2007). This lower transpiration rates at e[CO_2_] are common for both C_3_ and C_4_ plants (Ainsworth and Long, 2005). Lower transpiration rates under e[CO_2_] may decrease the translocation of glyphosate from its site of application, to other parts of the plants (**Figure 2**). Several studies have shown that glyphosate resistance endowed by decreased glyphosate translocation in populations of *L. rigidum*: C_3_ weed (Lorraine-Colwill et al., 2003; Wakelin et al., 2004) and *S. halepense* (Vila-Aiub et al., 2012).

## Conclusion

In a summary, plant biochemical, physiological, and morphol ogical changes under e[CO_2_] may favor over the glyphosate resistance development in both C_3_ and C_4_ weeds in different extent. Based on the critical review of the literature, C_3_ weed species would be able to develop glyphosate resistant more easily than the C_4_ species under increased atmospheric [CO_2_]. There is a pressing need of unravelling the precise details of the biochemical, genetic, and molecular measures by which plants develop glyphosate and/or other herbicide resistance and how e[CO_2_] affects these measures. These details will help to wiser use of herbicides under changing climate conditions and develop the innovative integrated weed management strategies. Based on the current knowledge, it is wise to apply herbicides at recommended dosages under ideal environmental conditions, and to coincide with the most sensitive growth stages of weed plants to avoid further development of glyphosate resistant weeds.

## Author Contributions

NF made substantial contribution to concept, 80% of the manuscript writing, literature review and interpretation of the revision, and creation of the figures. SM made substantial contribution to manuscript writing. SF and BC made substantial contribution to manuscript editing and contribution to the concept. SS made substantial contribution to manuscript editing, concept, and create the figures. All authors gave final approval for the version to be submitted.

## Conflict of Interest Statement

The authors declare that the research was conducted in the absence of any commercial or financial relationships that could be construed as a potential conflict of interest.
